# Engineering Virtuous health habits using Emotion and Neurocognition: Flexibility for Lifestyle Optimization and Weight management (EVEN FLOW)

**DOI:** 10.3389/fnagi.2023.1256430

**Published:** 2023-11-22

**Authors:** Patrick J. Smith, Heather E. Whitson, Rhonda M. Merwin, C. Virginia O’Hayer, Timothy J. Strauman

**Affiliations:** ^1^Department of Psychiatry, University of North Carolina at Chapel Hill, Chapel Hill, NC, United States; ^2^Department of Medicine, Duke University Medical Center, Durham, NC, United States; ^3^Department of Medicine, Durham Veterans Affairs Medical Center, Durham, NC, United States; ^4^Department of Psychiatry, Duke University Medical Center, Durham, NC, United States; ^5^Department of Psychiatry and Human Behavior, Thomas Jefferson University, Philadelphia, PA, United States; ^6^Department of Psychology and Neuroscience, Duke University, Durham, NC, United States

**Keywords:** self-management, treatment tailoring, personality, executive functioning, psychological flexibility, self-regulation and self-systems theory

## Abstract

Interventions to preserve functional independence in older adults are critically needed to optimize ‘successful aging’ among the large and increasing population of older adults in the United States. For most aging adults, the management of chronic diseases is the most common and impactful risk factor for loss of functional independence. Chronic disease management inherently involves the learning and adaptation of new behaviors, such as adopting or modifying physical activity habits and managing weight. Despite the importance of chronic disease management in older adults, vanishingly few individuals optimally manage their health behavior in the service of chronic disease stabilization to preserve functional independence. Contemporary conceptual models of chronic disease management and health habit theory suggest that this lack of optimal management may result from an underappreciated distinction within the health behavior literature: the behavioral domains critical for initiation of new behaviors (Initiation Phase) are largely distinct from those that facilitate their maintenance (Maintenance Phase). Psychological factors, particularly experiential acceptance and trait levels of openness are critical to engagement with new health behaviors, willingness to make difficult lifestyle changes, and the ability to tolerate aversive affective responses in the process. Cognitive factors, particularly executive function, are critical to learning new skills, using them effectively across different areas of life and contextual demands, and updating of skills to facilitate behavioral maintenance. Emerging data therefore suggests that individuals with greater executive function are better able to sustain behavior changes, which in turn protects against cognitive decline. In addition, social and structural supports of behavior change serve a critical buffering role across phases of behavior change. The present review attempts to address these gaps by proposing a novel biobehavioral intervention framework that incorporates both individual-level and social support system-level variables for the purpose of treatment tailoring. Our intervention framework triangulates on the central importance of self-regulatory functioning, proposing that both cognitive and psychological mechanisms ultimately influence an individuals’ ability to engage in different aspects of self-management (individual level) in the service of maintaining independence. Importantly, the proposed linkages of cognitive and affective functioning align with emerging individual difference frameworks, suggesting that lower levels of cognitive and/or psychological flexibility represent an intermediate phenotype of risk. Individuals exhibiting self-regulatory lapses either due to the inability to regulate their emotional responses or due to the presence of executive functioning impairments are therefore the most likely to require assistance to preserve functional independence. In addition, these vulnerabilities will be more easily observable for individuals requiring greater complexity of self-management behavioral demands (e.g. complexity of medication regimen) and/or with lesser social support. Our proposed framework also intuits several distinct intervention pathways based on the profile of self-regulatory behaviors: we propose that individuals with intact affect regulation and impaired executive function will preferentially respond to ‘top-down’ training approaches (e.g., strategy and process work). Individuals with intact executive function and impaired affect regulation will respond to ‘bottom-up’ approaches (e.g., graded exposure). And individuals with impairments in both may require treatments targeting caregiving or structural supports, particularly in the context of elevated behavioral demands.

## 1 Aging, chronic disease management, and functional independence

One of most important challenges of aging is the ability to retain functional independence (Motamed-Jahromi and Kaveh, 2020). Many older adults report that preserving their ability to live independently and engage in activities of their own volition is one of the most important aspects of approaching older age. While functional independence is understandably associated with preserved cognitive abilities (Gamage et al., 2019) cognitive status in and of itself does not fully explain the capacity for some individuals to engage in vibrant, independent activities in their older age (Molenaar et al., 2023). Indeed, although the clinical definition of dementia is defined by impairments of activities of daily living (ADLs), a substantial number of individuals without dementia experience loss of independence and associated reductions in quality of life (QoL) (Colón-Emeric et al., 2013). Loss of independence also results in and exorbitant amount of public health expenditures, as many individuals not only lose their own ability to meaningfully contribute to society, but also usurp time and other tangible resources from family members who provide their care (Falck et al., 2022). Better understanding the predictors and associated intervention targets to retain independence is therefore an important public health problem (Morgan et al., 2019).

Emerging data suggest that chronic disease management is a major driver of successful aging and retaining functional independence. As the population ages, the number of individuals managing chronic medical conditions such as hypertension, obesity, cardiovascular, and cardiopulmonary disease has increased dramatically due to more successful treatment mitigating mortality rates for both cancer and cardiac diseases. Among chronic medical conditions that impact the population, obesity and hypertension are two of the major modifiable risk factors for loss of functional independence. Obesity and hypertension both increase the risk of numerous chronic conditions, including cognitive impairment, and higher levels of obesity in midlife are a robust predictor of cardiovascular disease, stroke, and excess death. Fortunately, these risk factors are highly modifiable through lifestyle modification among middle-aged adults, primarily managed through increasing physical activity, modifying dietary habits, and reducing caloric intake for weight maintenance. Individuals who are physically active during middle-age have approximately 40% lower risk of Alzheimer’s disease and related dementias (ADRD). Similarly, interventions for both weight loss hypertension have demonstrated improved cognitive function following treatment, suggesting that more adequate management of behavioral risk domains may help to protect against cognitive decline: the strongest driver of functional impairment. Because of the multiple skills needed to effectively manage chronic diseases, multicomponent interventions have been shown to have the greatest efficacy for improving physical activity (Chase, 2015). As noted in a prior comprehensive review of long-term physical activity intervention programs, ‘a multipronged approach to interventions with goal of affecting behavior change that can influence multiple systems (physical, cognitive, and psychological health) … using a personalized approach [and personalized goals that incorporates] social support” is most likely to succeed (Chase, 2015). This review will highlight one such high-level systems variable that helps to determine successful aging and can be readily personalized: *self-regulation*.

The present review attempts to address a critical gap in the existing literature linking chronic disease management to long-term cognitive decline by integrating across several previously disparate literature bases: self-regulatory behaviors and health behavior maintenance, psychological profiles predicting health behavior changes (e.g., neurocognitive, personality, and affective phenotypes), and chronic disease predictors of functional independence. Prior reviews have been limited in their lack of integration across relevant literatures, relying on an examination of either single-factor predictors of behavioral management, focusing within single domains of function (e.g., psychological, cognitive, or social) without attempting to integrate across domains of function, focusing on either the initiation or maintenance of activity without an attempt to bridge these divergent targets, or by proposing limited, personalized approaches that do not cultivate sustainable lifestyle patterns. The present review attempts to integrate these previously disparate areas of study, proposing a more unified framework by which clinicians can approach personalization of treatment to enhance long-term behavior change maintenance.

## 2 Self-regulation and resilience through behavioral flexibility

Social and personality psychologists define self-regulation as the cognitive, emotional, and behavioral processes through which one establishes, monitors, and evaluates progress in personal goal pursuit (Karoly, 2010; Hoyle and Gallagher, 2015). Although prior work examining self-regulatory functioning in the context of aging has tended to focus on coping styles, an emerging literature suggests that flexibility in behavioral regulatory strategies is associated with better behavioral outcomes (Kato, 2020; Kato, 2021). Indeed, some individuals conceptualize psychological flexibility as a fundamental aspect of human health and effective behavioral coping with the inherent challenges of leading a fulfilling life (Kashdan and Rottenberg, 2010; Kashdan et al., 2020). Individuals with a broader set of coping strategies, greater variability in coping approach, and more effective coping skills demonstrate more effective behavioral changes over time. Notably, effective coping involves both the learning of new coping strategies and the ‘unlearning’ of others that have proven ineffective. The engagement in such behavioral learning and ‘unlearning’ involves inherent discomfort and broadening of behavioral coping approaches, for which greater psychological flexibility and acceptance of discomfort in the service of learning are critically important (Tabibnia, 2020).

Psychological flexibility is inherently multifaceted and, accordingly, may be achieved through different psychological and behavioral changes across individuals (Ruork et al., 2022). Psychological flexibility involves an individual’s ability to adaptively respond to contextual demands in the service of valued actions, often by tolerating distressing experiences, being present focused, and cultivating flexible response patterns (Kashdan and Rottenberg, 2010). It may be immediately apparent from contemporary definitions of psychological flexibility that several important subprocesses facilitate higher-order flexibility in responding. Individuals who lack clarity in their values, who are avoidant of aversive internal experiences, who have trouble staying present focused, and who exhibit a narrow range of behavioral responses may all be viewed as inflexible, albeit in different ways. Because all behavioral responses may be judged effective or not based on context-related goals, examining specific patterns of behavior is sometimes less useful than assessing the capacity to generate flexible response patterns when needed. Examples include the use of amplifying anger, which may be injurious in some interpersonal relationships but necessary when confrontational interactions are warranted in some work-related settings. Similarly, the flexible generation of coping strategies may be beneficial for individuals who respond in a rigid manner but function to reduce mindfulness for individuals already disconnected from important interpersonal relationships. Much of this work and associated randomized trials was developed by Lynch and colleagues Multiple types of coping are required to adapt effectively to aging and cultivating different coping strategies may take different forms for different individuals (Lynch, 2018; Lynch et al., 2020).

Perhaps not surprisingly, recent conceptual frameworks examining psychological resilience have begun to focus on the importance of regulating affect as a critical component of learning new coping skills and psychological resilience (Tabibnia, 2020; Troy et al., 2023). Affect refers to the experience of feeling emotions and affective processing lies at the intersection of neurobiological and psychological factors (Troy et al., 2023). Individuals still early in the course of neurodevelopment exhibit an exaggerated affective response and difficulty regulating their affect (e.g., lowering arousal following a startle), as indicated by taking a longer time to self soothe (Kittel et al., 2017; Mamrot and Hanc, 2019; Stalnacke et al., 2019). Older adults, or adults who have experienced a neurological insult, also experience degradations in their ability to regulate affect but in a more variable and qualitatively distinct manner (Berry et al., 2019; Schweizer et al., 2019; Cotter et al., 2020). For many older individuals, their affective experiences are characterized by lower reactivity, a slower time course of increasing emotion (relative to dampening reactivity, as is the case with younger individuals), and more diverse and effective set of coping skills to modulate affect (Salehinejad et al., 2021; Stretton et al., 2022). As reviewed below, these changes track broadly with well-characterized of development and disruption to white matter pathways regulating frontal-subcortical pathways of affective control (Cotter et al., 2020; Shafer et al., 2020), most notably the interaction between areas within the salience and executive-control networks. Put more simply, affective differences broadly group into individuals who experience difficulties (1) up-regulating positive affect, (2) down-regulating negative affect, and (3) with a tendency to ruminate (Tabibnia, 2020). For clinical purposes, these can be thought of as differences that require (1) strategies to increase behavioral activation through up-regulating dampened areas within frontal-subcortical rewards systems pathways (Crowther et al., 2015; Alexopoulos et al., 2016; Douma and de Kloet, 2020; Tabibnia, 2020), (2) strategies to enhance the efficiency of relaxation, experiential acceptance, and distress tolerance that are mediated by limbic-PFC integrated brain structures (e.g., amygdala and insular cortex) (Fonzo et al., 2017; Hermann et al., 2017; Addicott et al., 2018), and (3) strategies to get individuals ‘out of their head(s)’ by increasing mindfulness, salience, and transcendence of self (Taren et al., 2017; Demos McDermott et al., 2019).

### 2.1 Individual differences in cognitive control and emotion regulation

As noted above, self-regulation encompasses both cognitive and affective mechanisms by which individuals pursue their goals in the face of life’s challenges. Although often not integrated into the geriatric literature, there are well recognized differences in how cognitive control influences and motion regulation strategies. Individual differences broadly track with three types of cognitive control: working memory updating, response inhibition, and set-shifting (Hendricks and Buchanan, 2016; Dreisbach and Fröber, 2018; Pruessner et al., 2020). From the neuropsychological literature, these cognitive control processes overlap at a population level but are often different at an individual level (Pruessner et al., 2020). Individuals with deficits in working memory may include adults characterized by inattention or distractibility. This differs from individuals who have response inhibition difficulties, who are typically characterized as more impulsive (Drossel et al., 2023). Similarly, distractible or impulsive individuals often differ from those with set shifting difficulties, who are often characterized as perseverative and exhibiting a ‘stickier’ personality style (Alexopoulos et al., 2015; Voon, 2015). Working memory and updating is more reliant on dorsolateral PFC areas, inhibitory control on the anterior cingulate and ventromedial PFC, and perseveration results from disruptions to posterior cingulate and dlPFC brain regions (Reineberg et al., 2022). In essence, the type of executive control difficulty may vary substantively between individuals and overlap with affective control brain networks (Langner et al., 2018), leading some thought leaders to propose that conceptualizing interrelationships between executive and affect control is best conceptualized as an integrated self-regulation system (Langner et al., 2018).

A related area concerns how individuals with different cognitive profiles tend to benefit differentially from distinct self-regulation strategies, including the use of emotion regulation vs. behavioral regulation. As reviewed in detail elsewhere (Pruessner et al., 2020), effective emotion regulation necessitates flexibility in the use of several different regulation skillsets: (1) strategy stopping or switching, (2) strategy maintenance, and (3) monitoring. Individuals who are less cognitively flexible may not use an adequately variable set of coping responses to help manage daily stressors, failing to either initiate new coping strategies or switch from ineffective ones (Kato, 2022).

Similarly, individuals who are more concrete or with reduced working memory lack the ability to accurately appraise whether a selected coping skill is working, so that effective strategies can be maintained and ineffective strategies modified or eliminated (i.e., referred to as the *shielding-shifting* dilemma) (Goschke and Bolte, 2014). Individuals who tend to engage in cognitive reappraisal strategies (e.g., thinking about their situation in new ways to reduce distress) may find it necessary to switch to distraction strategies in high-intensity emotional situations (Birk and Bonanno, 2016).

To give several widely studied interactions between cognition and affect for effective self-regulation, individuals with lower working memory abilities have a more difficulties engaging cognitive reappraisal (which requires substantial working memory) in order to regulate their affective responses (Schweizer et al., 2013). Not surprisingly, negative affect has a much greater overlap and impact on working memory updating than it does on other cognitive control domains (Price and Duman, 2020; Pruessner et al., 2020). Individuals who are more depressed, anxious, or avoidant tend to experience greater deficits in working memory than their ability to inhibit impulsive behavior (Saleh et al., 2017; Schweizer et al., 2018; LeMoult and Gotlib, 2019). Similarly, individuals with lower inhibitory control may have more difficulties inhibiting pre-potent impulses, particularly when they are experiencing acutely higher levels of psychological distress, sleep deprivation, or physical fatigue (Hall and Fong, 2015; Evans et al., 2016).

### 2.2 Age-related differences in self-regulatory styles

**Figure 1:**
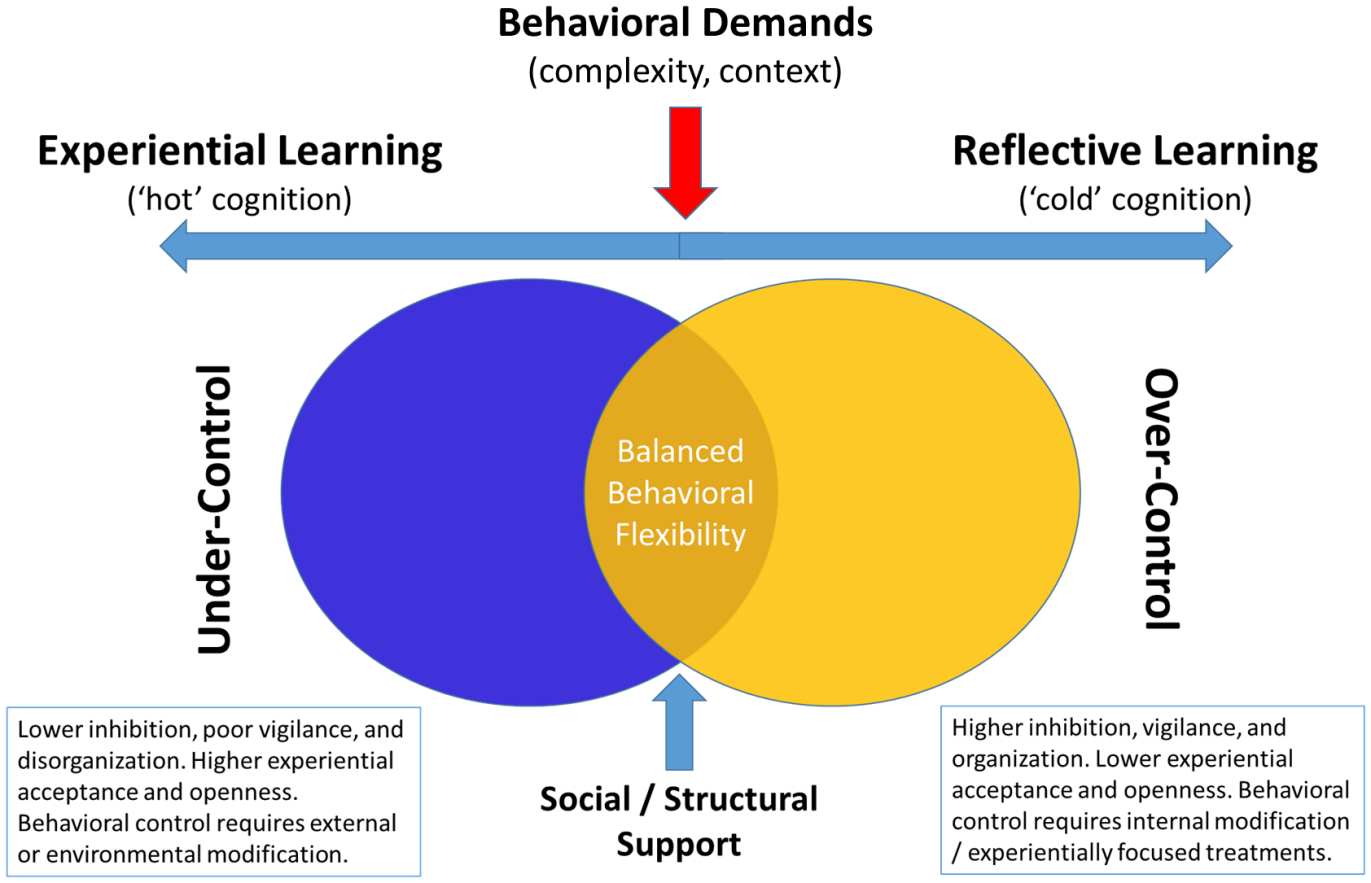
Conceptual diagram linking cognitive and psychological profiles to behavioral over-*vs*-under control style.

While there is a substantial literature on each of the various components of self-regulation in healthy adults, there are surprisingly few discussions of how self-regulation changes as we age. Differences between older and younger individuals emphasize an important and under-characterized pattern of self-regulatory functions. Older individuals are often more effective in their coping responses due to a wider behavioral repertoire of coping skills, a greater depth of experience using those coping skills, and an ability to use different coping skills across different contextual settings. Therefore, while they may experience some neurobiological inefficiencies in regulating affect, they are better able to adjust their behavior to compensate for this. Put simply, older individuals coping effectively are better able to regulate their behavior to meet contextual demands, exerting greater behavioral control when needed and more effectively using psychological acceptance approaches in situations where they have limited control. Individuals constantly using control-based coping approaches may have a greater likelihood of developing disorders characterized by ‘over-control’ (e.g., disordered eating, borderline personality disorder, obsessive compulsive disorder) (Ben-Porath et al., 2020; Baudinet et al., 2021; Isaksson et al., 2021), whereas individuals who fail to exert control when its needed may risk developing conditions characterized by disinhibition, poor behavioral regulation, or social disconnection (Burnell et al., 2022; Jones and Schuz, 2022; Wang et al., 2023). Ultimately, adaptive functioning requires flexible up- and down-regulating of self-control impulses depending on the context (Figure 1). A critical limitation in the existing literature is tailoring interventions in order to better understand how different phenotypic patient presentations impact how individuals more effectively modulate self-regulatory functions.

An important but underemphasized aspect of independent aging is the prognostic association between cognitive functioning and preserved self-management abilities (Mace et al., 2017). Although dementia is typified by impairments in memory, impairments in executive functioning are far more predictive of loss of functional abilities and impaired IADLs, even among individuals with comorbid memory impairments (Colón-Emeric et al., 2013; Mansbach and Mace, 2018). Notably, an emerging body of evidence suggests that elements of psychological functioning, including personality factors and associated emotion regulation abilities, interact with cognitive functioning to predict independence (Roy et al., 2016; Brothers and Suchy, 2021). For example, individuals who exhibit greater trait levels of openness to experience and lower levels of neuroticism appear to better navigate the complexities of aging compared to their counterparts (Williams et al., 2010; Hayward et al., 2013; Dermody et al., 2016; Mertens et al., 2022). Similarly, individuals who more frequently engage in ineffective coping behaviors, which may deplete self-regulatory capacity, show greater executive functioning lapses during daily life (Brothers and Suchy, 2021). Taken together, it is likely that impairments in executive functioning interact dynamically with elements of emotional functioning to influence older adults ability to successfully retain independence.

Understanding the interaction between cognitive capacity and psychological functioning is particularly important because executive functioning is critical both for preserving independence and to optimal engagement in health behaviors protecting the brain from future decline in cognitive abilities (Daly et al., 2014; Allan et al., 2016). Habitual engagement in physical activity, healthy dietary habits, medication adherence, and vascular risk reduction are all strongly influenced by the ability to regulate one’s behavior (e.g., self-organize, inhibit untoward impulses, self-motivate) (Reimann et al., 2020; Appelhans et al., 2021). In a recent review of prospective studies linking executive function to health behaviors, the authors reported that executive function deficits were linked to every health behavior outcome (e.g., physical activity, dietary health, medication adherence, tobacco use, etc.) with wide variation in the particular executive function component examined (Reimann et al., 2020). For example, although inhibitory control was frequently linked with poor health behaviors, cognitive flexibility, working memory, planning, and problem-solving were also predictive for other behaviors. Recent data has suggested that the three key subdomains of cognitive function representing critical capacities for maintaining healthy lifestyles on an individual level are inhibitory control, cognitive flexibility/shifting, and updating/working memory (Allan et al., 2016).

A critical component of effective coping involves behavioral flexibility, which is closely related to, and partially constrained by, levels of executive function (Kato, 2021). For example, even among individuals without cognitive impairment there is wide variation in their premorbid levels of cognitive flexibility, inhibitory control, and updating/working memory (Miyake et al., 2000a,b). Lower levels of functioning in any of these subdomains has been linked to worse health maintenance behaviors, including medication adherence (Stilley et al., 2010), weight management (Gowey et al., 2021), and physical activity (Hall et al., 2008). Even subclinical, relative weaknesses within these three subcomponents of executive function alone can result in individuals who will tend to show response patterns notable for being more concrete, impulsive, or distractable, all of which intuit different methods of management (e.g., coping skills training with strategy development, environmental modification, and use of reminder systems). With age-associated decrements in cognition, the ability to flexibly cope with new situations may be constrained as these skills become less efficient (Park and Reuter-Lorenz, 2009; Oberlin et al., 2016; Aschenbrenner et al., 2023), creating a potentially ‘vicious cycle’ by which worsening executive function leads to poorer behavioral regulation, which further degrades executive functioning resources (Morris et al., 2015; Grech et al., 2019).

### 2.3 Regulating behavior over time: temporal self-regulation and lifestyle maintenance

As the sections above demonstrate, there are many aspects of how self-regulation changes during the aging process that have yet to be fully delineated and applied. A critical gap in the current literature linking chronic disease management to functional independence is the lack of sustained lifestyle maintenance over time (Rhodes and Sui, 2021). The vast majority of individuals have difficulty maintaining behavior changes over time, such as sustaining physical activity levels (Lachman et al., 2018). Many individuals show chronically sedentary levels of activity and even among those who engage in a lifestyle intervention program, ≥75% tend to show reversion to their sedentary habits over time (Graham et al., 2020). Rates of poor lifestyle maintenance are also found among individuals who report motivation and intentions of engaging in exercise and dietary change, suggesting that these low rates are not exclusively due to lack of motivation (Huang et al., 2020). Indeed, this widely recognized discrepancy between intentions and action has been discussed in detail elsewhere, but appears partially attributable to individual differences in neurobiological factors and also to wide differences in contextual and environmental characteristics that act as facilitators or barriers to optimal engagement (Paganini et al., 2022; Dorina et al., 2023; Wang et al., 2023).

An emerging framework with both predictive and explanatory potential for the lack of lifestyle engagement across different groups is Temporal Self-Regulation Theory (Liddelow et al., 2022; Wang et al., 2023). This theory incorporates both psychological factors and neurobiological factors to explain high variability in success with lifestyle changes. Specifically, TST incorporates information on health behavior beliefs and current intentions with both historical data on pre-potency of habits and self-regulatory capacity as influencing ultimate behavioral outcomes. Components studied within this theoretical framework include the pre-potency for activity engagement, the degree to which the behavior has a prior habit formation, and the degree of executive functioning, which impacts both inhibitory control and also self-regulation of behaviors of differing complexities and across different contextual demands (Dorina et al., 2023). For example, behaviors of greater complexity or that are being engaged in within new contexts are inherently more prone to lapses. Due to this greater amount of ‘behavioral friction’ (Wood and Neal, 2016), it is not surprising that only highly motivated individuals are successful in adopting new, complex behaviors across contexts (Dorina et al., 2023). In addition, prior meta-analytic and clinical studies (Wang et al., 2023) have demonstrated that greater age and poorer executive function moderate the association between intentions and action, with individuals of greater age and lower executive function demonstrating greater discrepancies between their intended behaviors and actual actions (Dorina et al., 2023).

The importance of temporal self-regulation may be particularly important for aging adults, many of whom may be attempting to adopt new health habits following a new medical diagnosis or related health concerns. Many individuals actively engaging in healthy lifestyle habits, or with a prior history of such practices, will be more effective in their adoption of new behavioral repertoires in order to cope with their new chronic medical conditions. For these individuals, providing structural support in the form of supervised rehabilitation or peer-support maybe more than adequate to help facilitate their success with reengaging in health behaviors. In contrast, for individuals being encouraged to learn new health habit behaviors they will by definition be asked to engage in (1) complex behaviors that are (2) in different contexts that they are not familiar with. Perhaps not surprisingly, the most common time for dropping out of lifestyle intervention protocols is within the first few weeks of treatment, when participants are being asked to make the most active and disruptive changes (Bentley et al., 2021; Collins et al., 2022). In parallel, individuals with greater comorbidity burden (Heydarpour et al., 2015; Viken et al., 2019) and lower levels of self-efficacy are the most likely to drop out prior to completing rehabilitation, during the initiation phase of behavior change (Collins et al., 2022). In contrast, individuals with poorer executive function, lower levels of support, and less access to structural resources are at greatest risk for failing to maintain behavior changes over time, regardless of their initial success (Annesi et al., 2016; Mazzoni et al., 2021; Paganini et al., 2022). For this reason, recent reviews on physical activity maintenance stress the importance of flexible response patterns over time and dynamic coping approaches as critical predictors of maintenance (Rhodes and Sui, 2021). Similar findings have been reported for weight loss maintenance, with both psychological (e.g., self-efficacy) and cognitive factors facilitating self-regulatory behaviors serving as important predictors of ultimate success (Varkevisser et al., 2019; Eykelenboom et al., 2020; Roordink et al., 2022). In contrast, basic demographic factors (e.g., age, biological sex, race/ethnicity, socioeconomic status, etc.) were not predictive of weight maintenance (Varkevisser et al., 2019).

The importance of maintaining intact cognitive functioning is particularly relevant for older adults seeking to maintain health lifestyle changes, as emerging evidence suggests a bidirectional relationship between executive function and lifestyle engagement, creating a ‘virtuous cycle’ of increased physical activity and neurocognition (Daly et al., 2014; Allan et al., 2016). Although causal associations between cognitive factors and behavioral outcomes are complex and likely multifactorial (Danks and Davis, 2023), available evidence strongly supports the assertion that better cognitive function predicts healthier lifestyle maintenance, that lifestyle predicts better cognitive function, and that, even in the context of randomized trials lasting several months, treatment-related cognitive changes may prospectively predict behavior changes. Specifically, (1) individuals with greater executive function demonstrate greater capacity for maintaining healthy lifestyles over time (Gettens and Gorin, 2017; Kulendran et al., 2017; Audiffren and Andre, 2019; Butryn et al., 2019; Pfeffer and Strobach, 2021; Walo-Syversen et al., 2021), (2) healthier lifestyles (e.g., greater physical activity and lower obesity) associate with higher executive functions prospectively (Eskes et al., 2010; Best et al., 2015; Lu et al., 2016), and (3) treatment-related improvements in executive function predict greater maintenance of behavior changes over subsequent follow-up assessments (Baruth and Wilcox, 2014; Best et al., 2014; Allan et al., 2016; Audiffren and Andre, 2019). The Taken together, existing evidence suggests that intact executive functioning could be conceptualized as a necessary but not sufficient component of behavioral self-regulation (Allan et al., 2016; Gettens and Gorin, 2017; Butryn et al., 2019; Reed et al., 2020; Pfeffer and Strobach, 2021; Szabo-Reed and Donnelly, 2021).

### 2.4 Personality and self-regulatory behavior change techniques

Self-regulation engages a number of stable individual differences or personality variables. For example, using the five factor model of personality (openness, conscientiousness, extraversion, agreeableness, and neuroticism), personality facets demonstrate consistent associations with broad health behaviors in population studies (Bogg and Slatcher, 2015). Individuals who are more conscientious and less neurotic have been shown to have lower risk of mortality and developing chronic medical conditions, although the linkages between personality and health outcomes continue to be elucidated. Recent work suggests that is likely personality and health outcomes is linked through ‘assimilative’ processes, with personality factors tending to predict behavioral response pattens and the likelihood of influence by situational facilitators/barriers (McCrae and Sutin, 2018). For example, individuals who are more neurotic and less open to experience exhibit higher levels of anxiety sensitivity (Wauthia et al., 2019) and experiential avoidance (Mohammadkhani et al., 2016), which may lead them to engage in avoidant behavioral patterns (Sirois, 2015; Sirois and Hirsch, 2015).

A large and diverse literature base suggests that individuals are naturally inclined toward different though common behavioral coping processes (Carver and Connor-Smith, 2010). These can be broadly grouped into action oriented or active coping or avoidant coping through behavioral disengagement of varying forms. Individuals who are more conscientious and open to experience tend to cope in a more approach-oriented fashion. In contrast, individuals with higher levels of neuroticism are more likely to disengage and engage in avoidant coping (Mohammadkhani et al., 2016), and as a corollary, individuals who are more agreeable and conscientious are less likely to disengage (Carver and Connor-Smith, 2010), though may differ in the degree to which they pursue action-oriented coping strategies. Similarly, trait-level individual differences in affective intensity and positivity predispose individuals toward a greater tendency toward behavioral control, behavioral approach tendencies, and avoidance (Segerstrom and Smith, 2019). In parallel, studies of individual differences in self-regulatory function suggest three broad behaviors most closely associated with ‘persistence’ (Moshontz and Hoyle, 2021), each facilitated by overlapping but distinct brain networks. As described by Hoyle and colleagues, these include ‘resisting, recognizing, and returning.’ These track closely with the three cognitive control domains noted above (inhibitory control, cognitive flexibility, and working memory/updating). These neurobehavioral functions in turn are typically associated with differential aspects of the frontal-subcortical brain networks, with inhibitory control tracking closely with SN regions including the ACC (Tuulari et al., 2015), cognitive flexibility with posterior cingulate functions (Wan et al., 2015), and working memory associated with dorsolateral prefrontal cortex function (Mars and Grol, 2007; Lantrip et al., 2019).

In contrast to cognitive functioning, some aspects of personality tend to remain relatively stable over time, vary widely across individuals, and have been associated with different elements of executive functions (Rike et al., 2018; Kemps et al., 2020; Van Malderen et al., 2020). Differences in personality also intuit different behavioral intervention approaches (Ma et al., 2021; Alqahtani et al., 2022). Individuals with higher levels of conscientiousness tend to exhibit better planning and organizational abilities, are more vigilantly engaged with healthy lifestyle habits, and tend to engage in more self-monitoring behaviors. However, this relationship may be constrained among individuals who are also highly neurotic and you have lower levels of trait openness, for whom a more ritualized and rigid approach to coping may develop overtime (Bonanno and Burton, 2013). Conversely, individuals who have higher levels of trait openness may engage in more flexible coping patterns. But in combination with low levels of conscientiousness they may fail to adequately plan for or update these coping approaches for workability within changing life circumstances and therefore fail to adequately utilize them. It is therefore important to consider both personality and cognitive abilities when considering effective behavioral changes.

**Figure 2:**
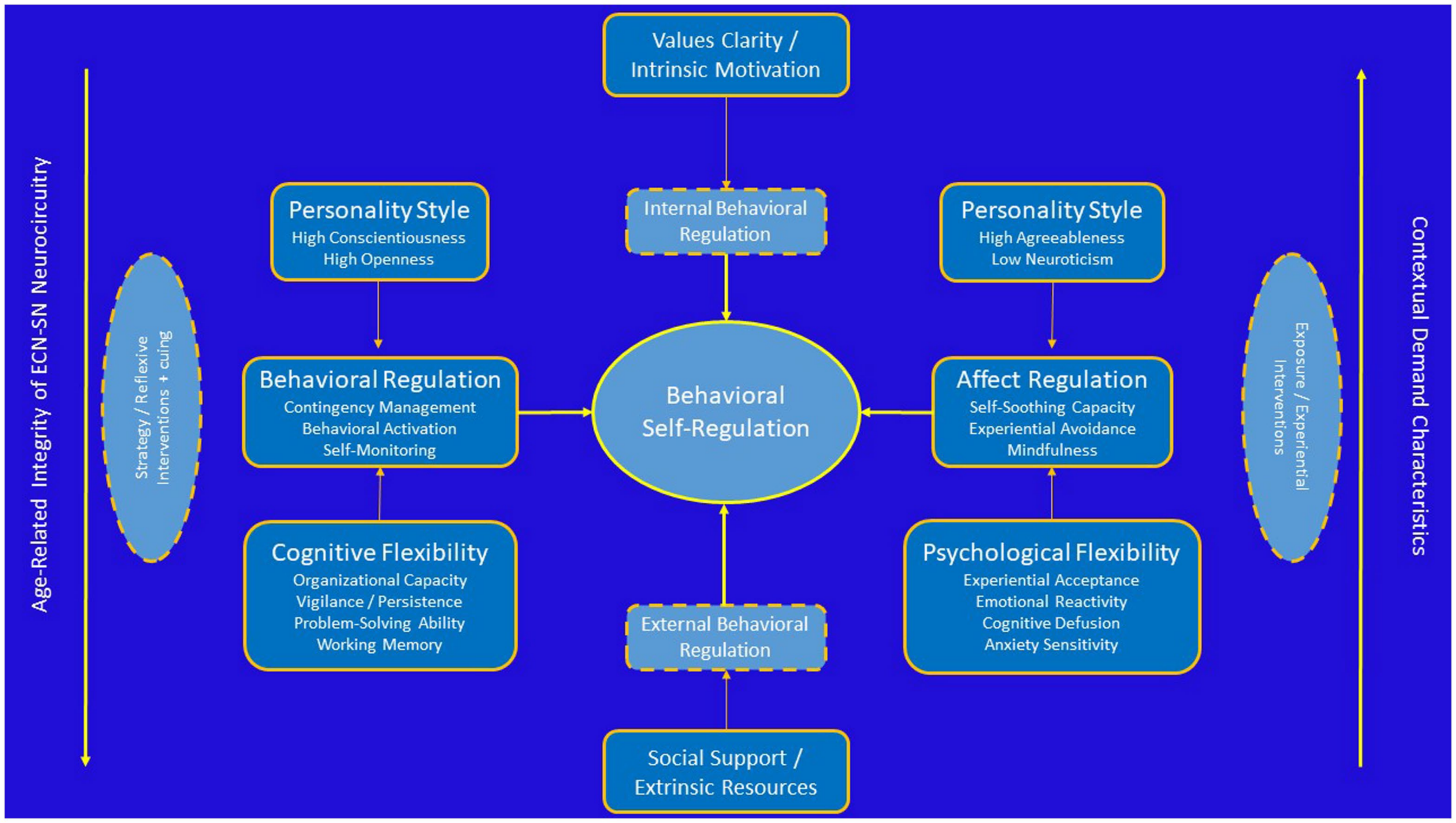
Conceptual diagram linking individual differences in personality and cognitive function to regulation of health behavior changes.

As noted earlier, individuals may be naturally predisposed toward several different action tendencies and these likely have a strong evolutionary intuition. Individuals who are more neurotic and have less openness to experience may be less likely to make major behavioral changes that require them to engage in what is perceived as riskier behaviors out of their comfort zone. For individuals who could benefit from weight loss, this often means greater friction embracing new behavioral patterns at the expense of familiarity with older ones, however ineffective. Similarly, Individuals more open to experience and conscientious may be more effective at making large behavioral changes with the recognition (through self-monitoring) that such changes are needed. In both cases, behavior could likely have an impact on the ability to make behavioral changes in response to changing health-related contingencies. At the same time, some elements of personality may adversely impact the ability of an individual to organize their lives in the service of self-management toward behavioral goals. Among chronic disease populations, individuals who are more conscientious, agreeable, open to experience, and less neurotic are more likely to maintain behavioral changes relative to their counterparts (Jerant et al., 2009, 2010, 2011). In addition, individuals exhibit more control over their own behavioral routines (Knittle et al., 2020) also tend to experience greater affective benefits from health behaviors, particularly physical activity (Ekkekakis et al., 2005). Such phenotypic measures, while not directly associated with personality variables, may provide important predictive information to tailor preventive interventions (Bryan et al., 2017). Emerging data suggest that affective responses are the critical mechanism linking diverse factors such as personality and cognitive functioning to long-term health outcomes (Leger et al., 2021) and that affective responses to physical activity are a robust predictor of physical activity maintenance (Figure 2; Boyle et al., 2019; Pfeffer and Strobach, 2021; Dunton et al., 2023). Taken together, available evidence increasingly points to the need for understanding of functional mechanisms by which behavior change techniques associate with health behavior changes, including an examination of interactions across external, internal reflective, and internal affective domains (Michaelsen and Esch, 2022).

### 2.5 Neurobiological underpinnings of self-regulation

The construct of self-regulation has been widely used by cognitive neuroscientists to understand complex patterns of neural activity in both healthy and impaired populations. Self-regulatory capacity appears to overlap systematically with markers of frontal-subcortical integrity, efficiency of network activation within the salience (SN) and executive control networks (ECN), and with behavioral markers of executive function (Stubberud et al., 2020; Fournier et al., 2021; Kim and Han, 2021; Smith and Merwin, 2021). Available evidence suggests that preserved cognitive flexibility through balance of highly interconnected cortical systems is critical for resilient self-regulatory function, with an anterior locus critical for behavioral implementation activation (SN) and a posterior locus critical for cognitive reflection and behavioral evaluation (ECN). In addition, the ability to self-regulate affect in the service of implementing behavioral response patterns appears to rely on critical interconnections between default mode network (DMN) and SN brain regions responsive to contextual cuing and experiential training (Kaiser et al., 2016; Walsh et al., 2019; Nagy et al., 2020; Pisoni et al., 2021). Among individuals with behavioral phenotypic evidence suggesting dysfunctional, *hyporesponsivity* within reward-system pathways (e.g., anhedonia, psychomotor slowing, low vigilance, poor sequencing), behavioral activation approaches appear most effective (Detloff et al., 2020; Nagy et al., 2020). Among individuals with phenotypic evidence suggesting *hyperresponsivity* within reward-system pathways (e.g., elevated startle response, ruminative, experientially avoidant), experiential interventions with an exposure component appear most effective (Hermann et al., 2017; Fitzgerald et al., 2018; Stojek et al., 2018). It has been widely documented that hyperactivation within SN brain regions strongly associated with diverse anxiety disorders and elevated psychological distress (Rabany et al., 2017). Alterations in DMN connectivity, in contrast, have been widely demonstrated among individuals with depression, pathological rumination, and ADHD, among other disorders. Finally, alterations within the ECN have been implicated in a multitude of clinical disorders, including mild cognitive impairment (Xu et al., 2020). Importantly, *the ability to shift behavior flexibly in response to contextual demands and update behavioral planning appears to track closely with interactions within the SN and ECN systems*, with hyperactivation resulting in behavioral rigidity, hypoactivation resulting behavioral disengagement, and behavioral regulation resulting from efficient and context-dependent shifting between both systems.

Notably, these distinct neural phenotypes track closely with widely known early neurodevelopmental differences among young children that track across three primary dimensions: emotional intensity, effortful control, and extraversion (Cooke et al., 2017; Brainstorm et al., 2018; Salvatore et al., 2020; Ip et al., 2021; Karlsson Linner et al., 2021; Munn-Chernoff et al., 2021). Emotional intensity (e.g., neuroticism) encompasses trait-level predispositions to experience emotions more intensely than others. Effortful control (e.g., executive function) encompasses a child’s ability to sustain attention over time, their capacity for impulse control, and their predisposition toward mind-wandering and internally directed thought processes (e.g., rumination). And finally, extraversion is the child’s predisposition toward seeking social relationships. These findings track with evidence from pediatric, adolescent, and young adult studies demonstrating elevated SN activity and resultant hyperconnectivity between areas in the SN-DMN associate with higher levels of neuroticism. Similarly, greater activation patterns within the ECN and connectivity between the SN-ECN appear to associate with higher effortful control levels.

There are at least two useful extensions of behavioral interventions and phenotypic variations within them that can be Better appreciated using this overarching framework as a conceptual reference. First, several of the areas within the SN and ECN systems show wide variation both in their initial myelination during development and in their normative disruptions associated with aging. This has been referred to as consistent with a ‘last in, first out’ pattern of disruption, where areas that myelinate the latest in adolescent development Appear to also be the most sensitive to age-related changes, particularly those secondary to cardiovascular disease (e.g., hypertension, obesity, diabetes), which are also highly prevalent. As an example, Areas in the dorsal lateral prefrontal cortex with important projections to the anterior cingulate are last to myelinate and tend to associate behaviorally with inhibitory control, such that younger adults without fully developed connections in these circuits have greater difficulty with behavioral inhibition. In older adults, age-related disruptions within these circuits tend to associate with slower processing speed, even though cognitive strategies tend to remain robust. In other words, the benefits of younger adults in processing speed are offset by lack of behavioral strategies, whereas the opposite pattern is true in older adults. Second, because behavioral phenotypes can result from multiple different underlying causes, understanding core neurocircuitry that mediates behavioral changes, either with normative maturation or in the context of intervention training, provide crucial insight into behavioral intervention strategies.

Several other principles from behavioral training paradigms examining neuroplasticity mechanisms are also relevant to understand the role of behavioral training on brain changes. These have been reviewed in detail elsewhere and are summarized briefly here (Kleim and Jones, 2008). First, areas that activate together tend to develop reciprocal synaptic connections, more commonly referred to as the Hebbian principle that ‘what fires together, wires together.’ Second, brain circuits that go underused become disrupted or even atrophied over time (‘use it or lose it’) and can be improved through training-associated usage (‘use it an improve it’). Improvements in brain circuitry are not monolithic, however, and will occur over different time frames depending on age, premorbid neurological injury, and the timing of intervention. Third, several key elements of behavioral training appear to have an important impact on neuroplasticity changes, including the repetition and intensity of training, the specificity of training, and the presence of interfering behaviors. Finally, training in one modality may enhance or inhibit gains in other domains.

### 2.6 Intermediate phenotypes of cognitive and psychological function

How can this self-regulation perspective help to identify commonalities across aging individuals? Cognitive and psychological functioning may converge to result in different affective phenotypes (Tabibnia, 2020). For example, a recently proposed tripartite model of affective resilience suggests three different phenotypic profiles with preexisting vulnerabilities based on differences in efficiency within distinct neuroanatomic functional networks. Within this framework the three profiles broadly corresponded to coping styles that required (1) up-regulation of positive affect, (2) down regulation of negative affect, and (3) strategies to get ‘out of your head’ by transcending the self (Tabibnia, 2020). These broad behavioral phenotypes also correspond to specific neuroanatomical system targets, specifically (1) up-regulating mesostriatal reward system pathways, (2) down-regulating autonomic/amygdalar reactivity within limbic brain regions, and (3) down-regulating default mode network pathways. Similarly, these broad phenotypes intuit different biobehavioral treatment strategies, including but not limited to (1) physical activity, (2) exposure therapies, and (3) mindfulness-based approaches.

Although phenotypes of resilience can no doubt be augmented by biobehavioral treatment approaches, there is strong evidence that genetic predisposition explains a substantial amount of our phenotypic ‘set point’, tending to be present from an early age and tracking within families. Decades of work across multiple disciplines has robustly demonstrated that personality and cognitive functioning have a high degree of heritability and only modest levels of overlap (Malanchini et al., 2019; Friedman et al., 2021; Krueger et al., 2021). For example, studies examining heritability estimates for cognitive abilities facilitating intact inhibitory control, cognitive flexibility, and other executive functions suggest that this capacity has a very high degree of heritability (Friedman et al., 2008), much higher relative to heritability estimates for general intelligence, for example (Freis et al., 2022; Gustavson et al., 2022). Although various aspects of executive functioning are amenable to improvement with behavioral training modalities (e.g., working memory, inhibitory control), individual differences in executive functioning are nevertheless ‘tethered’ to premorbid capacity even after training, suggesting substantial variability across individuals in this critical behavioral domain.

Similarly, several canonical personality factors have a high degree of heritability, including trait levels of neuroticism and openness (Jang et al., 1996). Although the degree of heritability for personality traits is somewhat lower than that observed for executive functioning, this may partly obscure the degree to which core temperamental elements of personality development, such as anxiety sensitivity, may play (Wauthia et al., 2019). In both cases, evidence increasingly suggests a vulnerability model of genetic transmission, in which predisposing vulnerabilities in cognitive and psychological phenotypic markers confer risk of developing an array of subsequent clinical disorders and with adaptive functioning more broadly (Bagby et al., 2008; Mertens et al., 2022). Moreover, individual differences in trait levels of openness, conscientiousness, and neuroticism appear robustly predictive of health behavior engagement: individuals who are more conscientious, open to experience, and lower in neuroticism, are much more likely to exhibit consistent health behaviors relative to their more neurotic and experientially avoidant counterparts. While the specific behavioral elements of heritability remain an active area of research, the robust association between personality profile and behavioral health outcomes provides an opportunity for treatment tailoring.

The importance of personalizing health behavior interventions it is perhaps most evident when examining the great degree of heterogeneity in approaches to change health behaviors. Within the growing Science of Behavior Change (SOBC) framework, more than 90 different behavioral approaches were identified that could be further reduced to 16 clusters. Self-regulatory behaviors represented within these cores included self-monitoring, goal-setting, and ‘down-regulating negative emotions’ (Wilson et al., 2020). Notably, a three-pronged approach to behavioral regulation has been noted by personality researchers as well, underscoring the collective importance of resisting, recognizing, and returning to goal-related pursuits (Moshontz and Hoyle, 2021). While the importance of each components will be intuitive to behavior change success, they rely on somewhat distinct neuroanatomical systems and will be harder or easier to engage with based on personality profile. Effective goal setting, for example, is at least partly dependent on selecting from a diversity of behavioral approaches and therefore partly dependent on personality domains such as openness. Individuals who are more open may not necessarily be effective at self-monitoring, which is more dependent on trait levels of conscientiousness. And the ability to modulate emotional reactivity is known to relate to trait levels of neuroticism. These three broad behavioral clusters (resisting, recognizing, and returning) can also be broadly mapped onto activation patterns within SN, ECN, and DMN networks, underscoring the importance of distinguishing motivational from cognitive strategy deficits when developing behavioral interventions (Berkman, 2018a,b).

## 3 Behavioral training modalities for self-regulation

Self-regulatory functioning may be best understood as a transdiagnostic behavioral process given its importance in facilitating multiple, disparate behaviors among adults (Sloan et al., 2017; Romer et al., 2021). Broadly speaking, impairments in the capacity to effectively regulate one’s behavior (Cahn-Weiner et al., 2000; Kiosses et al., 2001; Best et al., 2015; Hall and Fong, 2015; Mansbach and Mace, 2018) or effectively regulate one’s emotion response (Gyurak et al., 2012; Etkin et al., 2013; Zhang et al., 2019; Cotter et al., 2020; Troy et al., 2023) in the service of personally valued pursuits may result in an array of bad outcomes (Sirois et al., 2015; Amlung et al., 2017; Kauffman et al., 2022a,b). Behavioral regulation may be conceptualized as having a critical reliance on executive function, with intact executive function serving as a necessary but not sufficient capacity that may change with aging (Cotter et al., 2020; Hoyt et al., 2020), chronic disease burden, or neurological insult (Rike et al., 2014, 2018). Multiple sub-domains of executive function have direct and intuitive influences on behavioral regulation, including inhibitory function, problem-solving ability, learning/behavioral ‘updating’ to environmental or contextual contingencies, shifting attention, and maintaining vigilance over time (Moshontz and Hoyle, 2021). Importantly, many of these skill sets rely critically on the integrated function of dorsolateral and posterior frontal lobe regions (discussed in detail below), which are important for reflective analysis, pattern recognition, and strategy selection. Individuals who cannot recognize and adapt to functionally similar behavioral barriers (e.g., diverse outcomes resulting from poor time management, goal-setting, or self-monitoring) will tend to repeat the same mistakes in a perseverative fashion. Deficits in problem-solving abilities therefore often result from deficits in the ability to mentally abstract, integrating personally-relevant visual information for the purposes of self-monitoring, and generate alternative strategies to deal with complex problems. Such deficits are often treated using reflective strategies, goal-setting approaches, and collaborative self-management strategies (e.g., top-down). In addition, recognizing impulsive tendencies and resisting impulsive behavioral responses typically involve recognizing the cues and using cognitive control strategies for re-interpretation, again suggesting top-down strategy use that is partly contingent on accurate monitoring.

In contrast to more analytical, reflective approaches, several aspects of self-regulation involve persisting in the face of adversity. While these behavioral responses may be subsumed under executive function, these typically involve subdomains that include vigilance and effective sequencing of information (e.g., re-engaging repeatedly). Maintaining behavioral vigilance is typically facilitated by bottom-up behavioral strategies, which may involve sustained maintenance of undivided attention and self-motivation strategies to maintain engagement. Because these behaviors are subserved by anterior and paralimbic brain regions, including the anterior cingulate cortex, there is a high degree of emotional overlay facilitating persistence. Individuals better able to regulate emotional distress are therefore better able to persist in their goal pursuits.

Importantly, different aspects of self-regulatory function are leveraged by different intervention approaches. Many cognitive rehabilitation paradigms, for example, focus on strategy and process, whereas many ‘third-wave’ behavioral interventions focus on affect regulation through mindfulness and priming of personal values. One need not look far to appreciate the far-reaching importance of intact self-regulation, as impairments in self-regulatory capacity have been shown to predict substance abuse outcomes, medication non-adherence, impairments in activities of daily living, obesity, and impaired behavioral compliance writ large. A key conceptual distinction emergent from contemporary process-based therapies is the recognition that the ability to regulate one’s behavior is dependent on intact functioning within several critical domains, including the ability to intentionally flexibly modulate attention (‘cognitive flexibility’) and the ability to regulate or respond effectively to affectively distressing feelings (‘psychological flexibility’). Moreover, both processes are closely related to and impacted by a coherent underlying self-concept and personal values, which provide critical motivation for goal-related pursuits (Hayes and Hofmann, 2021).

Many contemporary therapies focus on only one of the domains above, implicitly assuming capacities in other domains that may constrain the efficacy of intervention approaches or sustained benefits. For example, Acceptance and Commitment therapy explicitly focuses on enhancing the salience of personal values, mindfulness, and behavioral response contingencies in the service of enhancing psychological flexibility but is relatively agnostic to the cognitive capacity of individuals. In contrast, intervention approaches among older adults with some level of cognitive impairment rely on a broader diversity of targeted domains, recognizing that cognitive control techniques, motivational factors, and affect regulation will all constrain an individual’s ability to volitionally modulate behavior in response to contextual demands. The widely used and efficacious Motivationally Enhanced Compensatory Cognitive Training for Mild Cognitive Impairment (ME-CCT-MCI) (Twamley et al., 2014, 2015; Sherman et al., 2017) provides a representative example. Self-regulatory function is a key psychological domain affected by multiple, disparate processes of change. As described in detail by Hayes and Hofmann (2021) self-regulation involves both overt behaviors and a cohesive, internalized sense of self. At the same time, important elements of self-regulatory skills, such as mindfulness, involve integration of affect, cognition, and attention, sometimes in the service of self-transcendence. Individuals who self-referentially perseverate due to a recent traumatic brain injury, for example, may subsequently exhibit increased depression that is being amplified by executive impairments that may improve. Self-regulation therefore combines all elements within contemporary, process-focused intervention initiatives (Hayes and Hofmann, 2021), including emotional, cognitive and attentional flexibility, perspective-taking and sense of self, personal values as a source of self-motivation, and construction of overt behavioral patterns based on these values. Critically, the flexible application of self-regulatory skills across multifaceted contexts in daily life place integral importance on individual differences in psychological flexibility (Kobylinska and Kusev, 2019), such that the ‘effectiveness of emotion regulation strategies depends on both situational contexts as well as individual differences in personality-like characteristics’ (Kobylinska and Kusev, 2019).

The ability to regulate one’s own behavior is one of the most critical psychological processes for leading happy, independent, meaningful lives. ‘Self-regulatory functioning’, or the ability to exert volitional control toward long term goal pursuits has been one of the most widely studied psychological functions, resulting in a wide and often contradictory literature base and multiple, closely related conceptual frameworks incorporating such concepts as executive functioning, self-management, grit, hardiness, and resilience. While there are important conceptual distinctions between each of these concepts, particularly in regard to their study within specific contexts, few overarching conceptual frameworks are available to help integrate these domains in order to inform personalized treatment approaches. An integrated framework is particularly important given the myriad factors plausibly affecting self-regulatory functioning across multiple levels of analysis, including variations in cognitive abilities across the lifespan, phenotypic differences in affective response patterns, and widely varying individual differences in contextual demands that will affect the importance and contingencies of individually initiated self-regulatory behaviors. The present, integrative review will attempt to provide a broad, parsimonious framework integrating elements across all three of these levels (neurobiological, affective, and contextual) toward a simplified framework to generate personalized intervention strategies.

The ability to effectively regulate one’s behavior relies on both functioning neurobiological systems within frontal-subcortical brain circuitry and the ability to effectively regulate ones affective responses to achieve valued behavioral goals. In addition, these functions have been shown to have differential vulnerability depending on contextual demands, with even small decrements manifesting themselves in the setting of high context demand characteristics. Similar to conceptual models for frailty, postoperative cognitive decline, or even more general biobehavioral models, the importance of efficient functioning within these behavioral domains is amplified in the setting of injury, elevated distress, or resource depletion (e.g., sleep deprivation, hunger, physical pain). While injury to systems integral for neurobehavioral facilitation of self-regulation are typified by changes in affect (e.g., traumatic brain injury, frontotemporal dementia, etc.), neurobehavioral and affective functions do not overlap consistently across individuals. For example, individuals characterized by over-control disorders (e.g., anorexia) often exhibit high levels of executive functioning or general intellect but may experience impaired ability to utilize these functions to regulate their behavior in the setting of dysregulated affect. Individuals more vulnerable to the effects of shame, for example, may exhibit impaired self-regulation of behavior even with superior levels of premorbid function. Similarly, individuals with age-associated cognitive weaknesses may better compensate for these with more effective coping skills, including strategies to regulate affect.

Self-regulatory functions represent a diverse array of complex behaviors, reliant on the integrity of multiple fronto-subcortical brain circuits (FSCs) (Lin et al., 2014; Friedman and Miyake, 2017; Munro et al., 2018; Baron Nelson et al., 2019) across broader integrated functions within the salience network (SN) and executive control network (ECN) systems (Menon, 2018; Bolton et al., 2020). Importantly, the functional connectivity between brain networks appears to degrade with age (de Dieu Uwisengeyimana et al., 2022), resulting in inefficiencies shifting between brain networks. Similarly, altered between-network functional connectivity associates with a diverse array of behavioral disorders (Ernst et al., 2019; Bolton et al., 2020; Kamiya and Abe, 2020), as well as self-regulatory functions within real-life contexts (Kronke et al., 2020). Such deficits appear to result from altered connectivity between SN brain areas and reciprocal functional connections in the ECN and DMN, which is not surprising given the critical role of the SN for a broad array of complex behaviors (Li et al., 2018). This may explain the preponderance of behavioral disorders emerging during periods within late adolescence and among older adults, both of which are typified by large, heterogeneous structural changes with frontal-subcortical brain circuits regulating affective and cognitive control (i.e., myelination and demyelination, respectively) (Goh and Park, 2009; Cooper et al., 2013; Park and McDonough, 2013; Reuter-Lorenz and Park, 2014; Bischof and Park, 2015). Moreover, available literature suggests a tripartite hierarchy of executive function components critical for preserved function: set-shifting abilities, working memory, and inhibition of prepotent processes (Kupis et al., 2021; Uddin, 2021). In other words, the ability to shift, update, and inhibit represent dissociable aspects of executive processes (Miyake et al., 2000b; Miyake and Friedman, 2012). These abilities are all integrally dependent on interconnections between SN and ECN brain regions (Kupis et al., 2021; Uddin, 2021), which has the highest density of dopaminergic receptors, in addition to other catecholamines.

*Contextual Demand Characteristics* Although often implicitly treated as uniform, individuals face vastly different challenges in their daily lives. Moreover, even within a given individual, it is not unusual for the demand characteristics of situational demands to vary dramatically over time. Examples include the need to handle increasingly complex job demands, to manage an increasingly burdensome and complex medical regimen for individuals with multicomorbidities, to balance caregiving demands of an aging parent with other role or occupational demands, or to experience the sometimes isolating challenges of aging without sufficient social support. Emerging evidence suggests that when contextual demand characteristics are taken into account, individual differences in executive functioning demonstrate a far more robust associations with lapses in behavioral regulation. These findings have been mostly widely reported within the Contextually Valid Executive Functioning (ConVExa) framework (Suchy et al., 2020). As an example, recent data has demonstrated that decrements in executive functioning were associated with medication non-adherence and that this association was amplified by overall medication regiment complexity (Liddelow et al., 2022), such that lower executive functioning predicted non-adherence as medical regimen complexity increased (Suchy et al., 2020). Emerging data also suggests that emotional control may moderate the effect of stress on biological markers of aging (Harvanek et al., 2021). Higher cognitive reserve capacity appears to play a buffering role against the adverse effects of social and environmental stressors (Cheval et al., 2019). Moreover, context stability may play an important role facilitating health behaviors independent of habit formation (Maher et al., 2021).

In addition to increasing contextual complexity, numerous modifiable psychological and behavioral factors appear to deplete self-regulatory capacity and worsen IADL performance among older adults at risk for loss of functional independence. Individuals who are prompted by situational stressors to more frequently inhibit expression of emotions (‘expressive suppression’) have been shown to have increased likelihood of impaired IADLs (Niermeyer et al., 2019; Suchy et al., 2019), and this vulnerability is further amplified by inconsistencies in daily executive function performances (Brothers and Suchy, 2021). In addition to depletion of emotional resources, poor sleep and increased pain interference (Tinajero et al., 2018; Niermeyer and Suchy, 2020) also appear to contribute to impaired executive functioning and IADL lapses in daily life. Similarly, higher cognitive reserve capacity appears to play a buffering role against the adverse effects of social and environmental stressors (Cheval et al., 2019). These findings are particularly important as evidence in older adults suggests that both cognitive flexibility and personality act in synergy to predict IADLs. Specifically, while both executive functioning, openness, and conscientiousness (Hall and Fong, 2013) were all important for preserved IADLs, conscientiousness and executive function exhibited synergistic effects on predicting impaired IADLs (Roy et al., 2016). Older adults also tend to preferentially select contexts less likely to require coping strategies that usurp cognitive resources.(Makowski et al., 2015) this may explain large individual variations in propensity to experience executive failures in particular contexts (DesRuisseaux et al., 2022).

The ability to effectively regulate one’s behavior relies on both functioning neurobiological systems within frontal-subcortical brain circuitry and the ability to effectively regulate ones affective responses to achieve valued behavioral goals. In addition, these functions have been shown to have differential vulnerability depending on contextual demands, with even small decrements manifesting themselves in the setting of high context demand characteristics. Similar to conceptual models for frailty, postoperative cognitive decline, or even more general biobehavioral models, the importance of efficient functioning within these behavioral domains is amplified in the setting of injury, elevated distress, or resource depletion (e.g., sleep deprivation, hunger, physical pain). While injury to systems integral for neurobehavioral facilitation of self-regulation are typified by changes in affect (e.g., traumatic brain injury, frontotemporal dementia, etc.), neurobehavioral and affective functions do not overlap consistently across individuals. For example, individuals characterized by over-control disorders (e.g., anorexia) often exhibit high levels of executive functioning or general intellect but may experience impaired ability to utilize these functions to regulate their behavior in the setting of dysregulated affect. Individuals more vulnerable to the effects of shame, for example, may exhibit impaired self-regulation of behavior even with superior levels of premorbid function (Dolezal and Lyons, 2017). Similarly, individuals with age-associated cognitive weaknesses may better compensate for these with more effective coping skills, including strategies to regulate affect (Chen et al., 2017; Titcombe-Parekh et al., 2018).

### 3.1 Contextual specificity: how self-regulation links emotion and cognition across contexts

Contextual demands are widely known to impact the number of coping resources and type of coping behaviors required to effectively cope with a given situation vary. At a broad level, this is widely recognized within the field of development, where age-associated developmental changes associate with gaining, broadening, and more tactical use different coping skills (Lougheed and Hollenstein, 2016; Lougheed et al., 2020). Similarly, individual difference studies of emotional coping strategies suggest that flexibility in emotion coping strategies and better use of specific strategies in the right context are the key elements explaining their efficacy (Kobylinska and Kusev, 2019). Individuals better able to use reappraisal strategies may cope more effectively in many antecedent-focused coping scenarios relative to suppressing emotional expression (Gross, 2002; Gross and Barrett, 2011; Gross, 2013), whereas acceptance-based strategies may work more effectively in some response-focused scenarios. Because emotions do not need to be regulated all the time but only when the interfere with desired behaviors or goals (Gross, 2014), differences in social or situational context make a substantial difference in the most effective strategy to select. Over time, as individuals encounter and are forced to cope with similar classes of stressors (e.g., relationship conflict, work demands, health concerns) they will either adapt to these new challenges by altering coping behaviors or retain rigid, ineffective strategies.

Examples for context-specificity encompass many lifestyle behaviors, including physical activity and dietary practices. For example, partaking in an unhealthier family meal may serve an important social function for important ceremonial activities (e.g., at a wedding or a funeral), but may result in numerous untoward health consequences if it becomes a habitual practice. This difficulty in being able to link specific behavioral responses to context is recognized as a major limitation of existing research (Aldao, 2013). Notably, available evidence suggests that change-focused strategies work best in situations that are more changeable, but work worse in uncontrollable situations (Haines et al., 2016). Understanding the *controllability of the stressor* is therefore important for selecting coping strategies.

*Intensity of contextual demand* is also an important component of understanding behavioral response characteristics. For example, prior work has demonstrated that the emotional intensity of context demands play a major role in predicting the type of effective coping response (Lindsey, 2020). Within the cognitive aging literature, greater cognitive load is well-recognized to play a role in coping strategies and distress levels (Nagamatsu et al., 2011). Dual-task paradigms of cognitive assessment, which introduce several task demands simultaneously to temporarily overwhelm cognitive load, are also more sensitive to early cognitive decline in preclinical adult samples (Whitson et al., 2018, 2021). Not surprisingly, ‘real-world’ behavioral demands also vary in their complexity and have been differentially linked to behavior change success. Individuals being prescribed a more complex medication regimen for the first time, for example, have greater difficulties if they have pre-existing executive function weaknesses (Liddelow et al., 2020).

A burgeoning area of research demonstrates that the association between executive functions and healthy behaviors are robustly influenced by emotional state (Schirda et al., 2016; Lantrip and Huang, 2017; Fitzgerald et al., 2018; King Johnson et al., 2023). Although cognition and emotion are often conceptualized differently, they share a substantial overlap in neuroanatomical representation and functional neuroanatomy, particularly within areas of anterior cingulate gyrus (Wang et al., 2008; Menon and Uddin, 2010). Moreover, it should be noted that in most cognitive tests are assessed under highly controlled conditions, such as with a technician in an outpatient office or using a computer tablet. While informative and highly prognostic, these tests may not always accurately represent functioning during the stressors of normal day-to-day functioning, where both the ability to modulate emotional responses and think strategically are critical for independent functioning (Nicosia et al., 2023). The distinction between ‘cold’ and ‘hot’ cognition has also been explored using executive functioning paradigms that incorporate an overlay of emotional control, such as when a participant has to respond under stressful circumstances or exert inhibitory control under pressure (Salehinejad et al., 2021). Tests such as the Iowa Gambling Task, the Wisconsin Card Sorting Task, and the emotional Stroop task are all examples (Verreckt et al., 2022).

Notably, executive functions as a class are strongly predictive of behavioral outcomes in ways that other domains of function (e.g., intelligence quotient) are not (Morimoto et al., 2016; Mansbach and Mace, 2018; Walo-Syversen et al., 2021). It also appears that deficits in EF associate with the largest differences across contexts, with some individuals exhibiting mild EF impairments demonstrating an outsized impairment in behavioral outcomes when contextual demands are higher, when behavioral control parameters are broader or lack clear contingencies, or when activities are less structured (Suchy et al., 2020; Park, 2021; Ho et al., 2022).

### 3.2 Optimizing behavioral outcomes using contextual hierarchies

A well-known feature of experientially focused behavioral therapies is their reliance on context demands to translate the wider use of skills learned in behavioral interventions into their daily lives. Common examples include exposure-based therapy for obsessive compulsive disorder, social anxiety disorder, and other fear extinction paradigms. Behavioral activation for depression uses similar approaches, in which value-based behavioral targets are titrated up over time using a hierarchical approach. Within each of these paradigms, the keys to success typically involve building confidence and self-efficacy for lower-level context demands then gradually increases the anticipated anxiety and/or context demands over time. Examples for someone with social anxiety who avoids shopping at the grocery store might be to first building up confidence by shopping at night, gradually moving the timing of shopping to busier times of day or adding complexity to the shopping trip (e.g., where they may need to ask for assistance).

Although individual differences in cognitive functioning are not often integrated within such paradigms, their importance is most easy to see for individuals undergoing driving testing. For teens or older adults going through driving assessments, they must first demonstrate basic skillsets such as stopping in response to a physical barrier, efficiently juggling cognitive demands for multiple subskills involved in operating the vehicle (e.g., pressing the pedal, steering, and operating the turn signal), anticipating other drivers intentions from context clues, etc. These skills are first assessed under low demands in order to ensure their competency. Subsequently the demand characteristics are gradually ratcheted up, moving from driving in a parking lot to a sparsely driven neighborhood, to a busy thoroughfare, and finally onto the highway.

Similar principles are used in the development and adaptation of training paradigms within sports and performance psychology. As individuals demonstrate high levels of competency for complex skills under minimal demands, they begin to be trained to use the same skills under increasingly higher levels of contextual demand that more closely parallel what they will encounter under ‘real-world’ scenarios. For musicians, this often begins by demonstrating competency for a skill by oneself, then with a small, receptive audience, then a larger audience, and finally a large and unreceptive audience. This ‘training under pressure’ is critical for broader use of learned skills.

In our own work, we have attempted to use similar principles to facilitate better uptake and use of behavioral skills in several ways. First, many of our behavioral interventions (predating the COVID-19 pandemic) prioritize the use of remotely-delivered treatments. By doing therapy with individuals often in their homes, contextualizing treatment within the environment they were living in, or in some cases at their places of work, we feel our therapies were better utilized and more robust to application. Second, we have put greater emphasis on ecologically valid assessment modalities to assist with better understanding how mood-related factors impact behavioral and cognitive changes during day-to-day life. Third, at least one of our recently funded trials is examining whether behavioral training conducted during times of higher context demands might result in better use of skills training. For example, in our current TEMPO trial (Time restricted Eating for Metabolic and Psychological Optimization; R61 AG080615-01) we are examining whether the effects of an intermittent fasting intervention on cognitive functioning are more robust during times of greater metabolic demand (i.e., the end of an 18-h fasting period).

Emerging data suggest that behavioral training in the setting of higher context demand increases the likelihood of ‘far transfer’ of skills into daily life. In a previous study of affective cognitive control capacity, individuals trained on an emotional working memory task demonstrated greater transfer of skills onto a ‘gold standard’ affective control measure, as well as greater efficiency of brain regions within the ECN (Schweizer et al., 2013). More recently, a pilot study of military veterans demonstrated that a cognitive-emotional training intervention improved, executive function, psychological functioning, and reduced activation of areas within the SN (Dolcos et al., 2021).

### 3.3 Social and structural support: moderators of self-regulation and keys to behavior change

Although a comprehensive review of social and structural supports on behavioral change interventions is beyond the scope of the present intervention (Kwasnicka et al., 2016), it is widely accepted that individuals with greater levels of support are more likely to make durable behavior change (Huang et al., 2009; Hunter et al., 2019). Indeed, some individuals place the role of social support networks as among the most instrumental aspects of behavior change maintenance, at times characterized as the ‘hidden social networks’ galvanizing more effective behavior modifications (Hunter et al., 2015). There are multiple reasons why better social support helps facilitate behavior changes including, but not limited to, (1) greater emotional support, (2) better instantiation of behavior changes through cuing use of coping skills across contexts, and (3) collaborative engagement and strategizing with someone familiar with person-specific challenges (Wood and Neal, 2016; Wood and Runger, 2016; Wood, 2017). As an example, following a neurological injury (e.g., stroke or TBI) individuals with engaged caregivers often have more opportunities to use compensatory skills across different contextual demands, consistent with the Multi Contextual Treatment Approach (Toglia et al., 2010; Toglia, 2011; Toglia et al., 2011; Nagelkop et al., 2021; Jaywant et al., 2022). In such circumstances, individuals benefit to a much greater degree from metacognitive strategies enhance transfer of behavioral skills, because their partner is more familiar with their style of thinking and therefore can better anticipate pitfalls (Toglia, 2011; Toglia et al., 2011; Foster et al., 2018; Arora et al., 2021; Nagelkop et al., 2021; Jaywant et al., 2022). Ongoing behavioral weight loss trials are attempting to delineate the specific roles for targeting (1) individual-level characteristics, (2) environmental facilitators of behavioral weight loss success, and (3) their potential synergistic effects (Cáceres et al., 2022).

Examples include incorporating booster sessions into the designing of exercise (Fleig et al., 2013) and/or weight maintenance treatments (Sawamoto et al., 2017), prioritizing group programs (Horwood et al., 2015) that actively integrate peer support approaches in the context of structured rehabilitation to promote maintenance of activity following the completion of formal training (Santiago de Araújo Pio et al., 2019), and incentiving rehabilitation staff members to monitor attendance and patient progress in order to enhance patient engagement (e.g., targeting the support structure level instead of the patient-level) (Bohplian and Bronas, 2022). In addition, a consistent theme in the extant rehabilitation literature is the finding that older, more medically complex, and more geographically isolated patients have the highest risk of both failing to complete formal rehabilitation programs and failing to maintain physical activity over time (Fleig et al., 2013; Heydarpour et al., 2015; Horwood et al., 2015; Heron et al., 2016). Approaches that pay greater attention to, and leverage available resources for, individuals with ≥ two of these characteristics (older age or cognitively impaired, greater medical complexity, and geographically isolated) will likely have greater success with patient outcomes and potentially reduced burden to the local medical system as a result. In the context of emerging telehealth rehabilitation initiatives (Santiago de Araújo Pio et al., 2019; Diamond et al., 2021).

**Figure 3:**
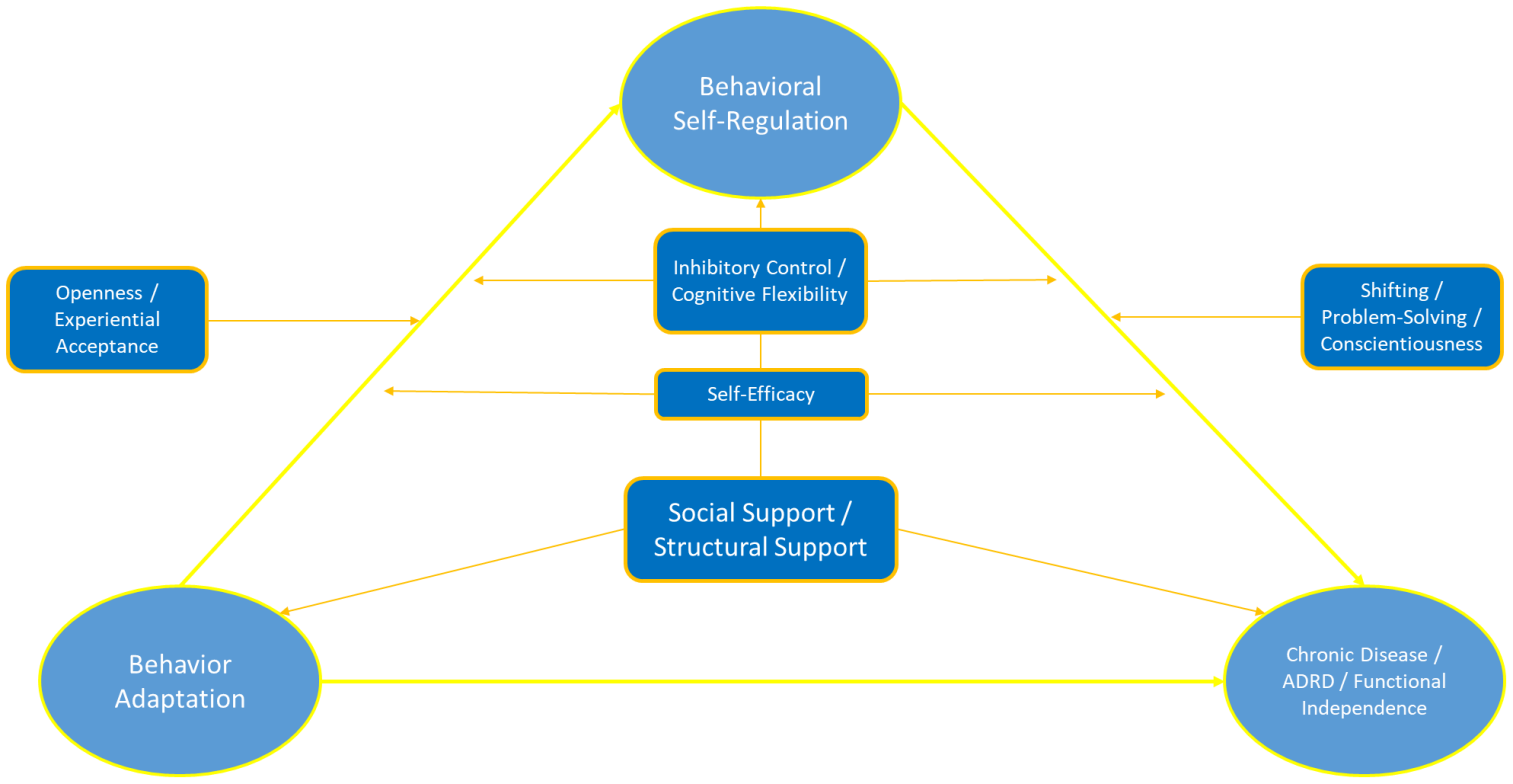
Psychological and cognitive domains at different intervention phases.

Greater levels of systemic or social support are likely important across both the treatment initiation and maintenance phases, but for different reasons. Adoption of complex behaviors such as physical activity as a new skill is inherently goal-directed and requires development and maintenance of new self-regulatory skills (Annesi, 2004; Bandura, 2004). Individuals with more complex disease processes notable for sedentary activity, such as COPD, are among the least likely to complete rehabilitation or maintain treatment gains after completion (Larson et al., 2014; Olson and McAuley, 2015). For such complex patients, available evidence suggests that self-efficacy is important for initial adoption of exercise behaviors but is often lower due to lack of familiarity with being physically active, anxiety, or avoidant coping (Marino et al., 2008; Watz et al., 2014; Spruit et al., 2015). Greater *social support during treatment initiation is therefore critical to facilitating learning the of target behaviors that are trained during rehabilitation*. In contrast to self-efficacy for treatment initiation, self-regulatory functioning is most strongly associated with maintenance of physical activity after rehabilitation (Olson and McAuley, 2015). As noted above, self-regulatory skills are often constrained by executive functioning capacity and are a critical determinant of treatment maintenance. Put simply, even after learning skills needed to sustain their health, many individuals will fail to use the skills during day-to-day life due to lack of organization, motivation, or ability to adapt to new challenges. Greater *social support during treatment maintenance is therefore critical to priming patients to use previously learned skills during daily life, assisting with reinitiation of training during periods of inactivity, and collaboratively engaging in problem-solving to adapt to new challenges*. Although available evidence suggests that well-designed programs are effective at increasing adoption of exercise (Covey et al., 2012; Mullen et al., 2012; Motl et al., 2013; Phillips and McAuley, 2013; Larson et al., 2014; Phillips and McAuley, 2014; Olson and McAuley, 2015; Spruit et al., 2015), few are effective in translating these gains initial gains into sustained maintenance (Larson et al., 2014), despite potential benefits to clinical outcomes with sustained physical activity (Figure 3; Coultas et al., 2018).

### 3.4 Empirical examples

In order to provide a brief empirical example demonstrating the potential utility of our proposed framework, we here provide additional analyses from baseline assessments of a recently completed intervention among individuals with resistant hypertension participating in a cardiac rehabilitation based behavioral weight loss program (Blumenthal et al., 2021) for whom both cognitive (Smith et al., 2019, 2022a,b) and psychosocial data were also collected (In Preparation) (Blumenthal et al., 2023). Although we found no correlation between global markers of executive function (Smith et al., 2022b) and negative affect (Blumenthal et al., 2023) (*r* = −0.004, *p* = 0.965), nor with self-efficacy (*r* = 0.04, *p* = 0.669) both domains contributed differentially to various behavioral health markers: actigraphy-assessed physical activity, 24-h urinary assessments of sodium/potassium, HbA1c, medication compliance (both MEMS caps and self-report), and dietary health assessed by the Dietary Approaches to Stop Hypertension Diet. Controlling for age, baseline systolic blood pressure, and total cardiovascular medication burden (daily defined dose) (Smith et al., 2022b) we found that both higher executive function (*B* = 0.23, *p* = 0.016) and lower levels of negative affect (*B* = −0.20, *p* = 0.029) associated with better medication compliance, whereas higher executive function associated with both higher potassium (*B* = 0.25, *p* = 0.006) and tended to associate with lower sodium levels (*B* = −0.17, *p* = 0.077), lower HbA1c (*B* = −0.22, *p* = 0.021), and higher levels of aerobic fitness (*B* = 0.19, *p* = 0.037). Higher self-efficacy associated with better DASH dietary adherence (*B* = 0.21, *p* = 0.022), lower HbA1c levels (*B* = −0.22, *p* = 0.014), and tended to associate with lower BMI levels (*B* = −0.16, *p* = 0.068). Explanatory analyses of the individual components explaining the executive function association revealed that several individual measures associated most robustly with better behavioral health habits, including measures of inhibitory control (Stroop Color-Word interference test), complex sequencing/shifting ability (Trail Making Test Part B, Ruff 2 & 7 Test), and working memory/updating (Digit Span test) (Smith et al., 2022b). These data help support the notion that both cognitive and psychological factors hold important information to understanding health behaviors and are worthy of additional study as to whether tailoring intervention approaches may facilitate better maintenance of lifestyle changes (Wang et al., 2022).

## 4 Phenotypic treatment examples

In order to appreciate how one might differentially approach therapy for individuals based on their phenotypic profile, several exemplars are presented below. These are based closely upon Individuals within our own randomized clinical trials examining the effects of lifestyle modification on cardiometabolic health. a primary example will first be introduced and then specific elements for phenotypic tailoring will be superimposed in order to equate background characteristics and clinical trial targets, while varying relevant process targets (Table 1). We also delineate between the treatment initiation and maintenance phases.

**Table 1:** Exemplars of potential tailoring based on intermediate phenotypes.

Psychological phenotype	Neurobehavioral profile	Behavioral change techniques and targets
*Psychologically flexible* High executive function/high emotion regulation (High conscientiousness/high openness/low experiential avoidance)	Organized, good cognitive flexibility, good working memory, balanced learning profile	*Self-monitoring*: flexible *Goal-setting*: utilizes SMART practices *Self-motivation/reinforcement*: flexible and efficient
*Single-minded* High executive function/low emotion regulation (Conscientiousness / Low Openness / High Experiential Avoidance)	Organized, good vigilance, good working memory, low cognitive flexibility, efficient learning slope, poor reversal learning (Salience network hyperconnectivity)	*Self-monitoring*: shift toward external progress markers and away from affective / somatic state. Experiment with different modes and delivery methods of self-monitoring cues to track progress and emphasize ‘workability’. Self-efficacy will be key to develop during the initiation phase, whereas cultivating broader and more diverse self-regulatory behaviors will be important in maintenance phase. *Goal-setting*: shift from distal to proximal targets (e.g., calories > weight loss). Experiment with different time-scaled metrics during maintenance phase of activity, such as yoking activity goals with major holidays or anniversaries. Consider varying within-week activity schedules to enhance flexibility while retaining accountability. *Self-motivation/reinforcement*: Link directly to values (e.g., playing sport with friend or talking with family member while walking [social], listening to podcast [education]), and other metrics that make justification for activity more values consistent. Targeting potential cognitive distortions regarding the rigid need for efficiency or being productive at the cost of being active for health.
*Anchored* Low executive function/high emotion regulation (Conscientiousness/high openness/psychologically flexible)	Disorganized, low vigilance, low working memory, constricted learning slope, perseverative (Salience network hypoconnectivity)	*Self-monitoring*: shift toward internal markers of enjoyment (affect > reflective). Limit to very few, easily deployed progress markers to emphasize reproducibility. Externally focused monitoring may be more beneficial, such as passive actigraphy measures. *Goal-setting*: shift to externally generated or time-bound goals. Keep more uniform weekly schedules of activities with less flexibility and higher routinization. External goals could also include passive monitoring through smartphone with reinforcement after hitting activity targets (e.g. 8,000 or 10,000 feet in a day). *Self-motivation/reinforcement*: Embed goals within highly routinized behavioral patterns. Link participation to external initiation mechanisms, such as a trainer, spouse, or TV program. Reduce behavioral friction to enhance transitions.
*Scattered* Low executive function/low emotion regulation (Low Conscientiousness / Low Openness / Psychologically Inflexible)	Disorganized, low vigilance, low working memory, poor reversal learning, perseverative (Salience/ECN network hypoconnectivity)	*Self-monitoring*: shift toward external structures and passive monitoring strategies that are not self-initiated. Provide feedback from passive monitoring to other individuals closely involved in supporting care or back to structural supports that can intervene (e.g. to primary care doctor, personal trainer, rehabilitation leader, etc.). Make all goals and updating external to individual. Build self-efficacy through early and frequent reinforcement. *Goal-setting*: shift to externally generated goals with low behavioral friction to engagement. For example, placing stationary bike in front of television prior to favorite shows coming on. Reduce the need for the individual to self-generate SMART goals and instead rely on external structural supports wherever possible, such as enrolling in group classes, peer support groups, or even using a personal trainer. *Self-motivation/reinforcement*: embed goals within highly routinized behavioral patterns. Link participation to external initiation mechanisms, such as a trainer, spouse, or TV program

As shown, tailoring of treatment approaches could vary between individuals who are (1) high in executive function and emotion regulation (‘psychologically flexible’), (2) high in executive function and low in emotion regulation (‘single-minded’), (3) low in executive function and high in emotion regulation (‘anchored’), or (4) low in both executive function and emotion regulation (‘scattered’).

Clinical Case Exemplar # 1 (60-year-old female attempting to lose weight).

*Example 1 (**Nimble**: high emotion regulation, high cognitive flexibility):* For the purposes of providing informative clinical examples that may impact tailoring of treatment approaches, we now take a very common case of a middle-aged woman trying to lose weight. Case examples are varied by personality and cognitive profiles to enhance differences along these phenotypic dimensions. First, a 60 year-old female attempting to lose weight who is high in conscientiousness, openness to experience, agreeableness, and executive functioning. For such an individual, most of the conventional approaches would be appropriate and effective. This individual would likely be able to efficiently learn and begin to incorporate behavioral changes into their life. For such an individual for whom the requisite aptitudes are already in place, it is likely that their prior experience with the target skill sets will be most relevant. For example, individuals who have never previously cooked healthy meals or who lack knowledge regarding nutritional content of their diet will need to spend time learning these content areas. However, it is likely that once they have learned these, their ability to recognize behavioral patterns, deploy appropriate response patterns, and adjust their own goals as needed should not be significant barriers. Given that this individual has the skills necessary to make behavior changes, two additional treatment elements have intuition, are implicitly more important, and should be explored in more depth: motivational factors and external environmental factors. If the individual is now seeking treatment, determining what motivational factor precipitated this and to leveraging this to motivate future behavior change is impaired. For example, if someone is now concerned because of being told their risk of a bad medical outcome was higher during a routine medical visit, utilizing a prevention-based approach that embraces risk mitigation may be helpful. In contrast, if an individual is motivated to feel better or to be able to fit in their favorite dress, a promotion-based approach may be indicated.

The most common area of deficit for individuals with the requisite skill sets to make effective behavioral changes is their lack of exposure to greater diversity of coping approaches or their lack of familiarity with the contextual demands they are now having to deal with. For example, many individuals are faced with stressors in which they experience a role change, such as having to be a caregiver for a family member and also now having to spend more time managing their own health concerns. Or from any cardiac patients, experiencing a heart attack may cause some degree of cardiac anxiety in which they may feel, for the first time, a lack of confidence related to their cardiac health and develop greater sensitivity to autonomic cues or an impulse to become hypervigilant regarding sympathetic nervous system changes. In such cases, providing opportunities for individuals to be exposed to a wider range of behavioral coping strategies are most beneficial. This may include the temporary provision of additional structure to help consolidate new skills, or to help reduce their role demands so that they can focus on rehabilitation (e.g., incorporating a part-time home health nurse to lower their own caregiving demands).

*Example 2 (**Single-Minded**: Low emotion regulation, high cognitive flexibility):* For the same individual who is higher in neuroticism and experiential avoidance, lower in conscientiousness, agreeableness, and openness to experience, the approach would differ significantly. First, the conceptual psychological processes of change would not be limited to providing information or structural support. Instead, the primary targets would fall under the frame of increasing *psychological flexibility*, which could include one or more of closely related components such as experiential avoidance, anxiety sensitivity, emotional dysregulation, and/or heightened shame sensitivity. These are individuals who have a high propensity to avoid distressing situations, will take longer to decrease their arousal in response to stressful situations, and will have a tendency to perseverate on previously used behavioral coping approaches out of familiarity and comfort, instead of tolerating the inherent stress of learning new ones. These are individuals with the skills to learn new coping strategies but who are hesitant to engage with them or accept needed changes but will do well learning new skills once avoidance has been reduced.

Importantly, the constituent components within this profile could be either neurovisceral in origin (e.g., anxiety sensitivity), resultant from anxious cognitive content (e.g., shame), or from associated lack of emotional coping processes (e.g., avoidant coping). It would be important first to identify what components within this individual’s profile are longstanding and dispositional, as could be obtained by asking about childhood temperament. Individuals who have always had a heightened startle response, greater generalized sensitivity to sensory stimuli, or show evidence of autonomic oversensitivity (e.g., vasovagal responses) might be more predisposed to biologically driven pathways of reactivity. In contrast, individuals with a history of PTSD or clinical conditions where anxious cognitions predominate might be viewed as having important contributions from personal background and psychiatric experience that could prove to be barriers. In either case, exposure-based approaches to increase psychological flexibility would be the primary treatment modality.

There are several reasons why this approach would be preferred over motivational or organizational kinds of approaches. First, motivational approaches generally rely on activation of salience network structures among individuals presumed to have problems resulting from under-activation. in other words, getting individuals to think more explicitly about their concerns, exploring discrepancies between an idealized self and current self, and an individual’s ownership of their health problems would likely increase anxiety leading to further avoidance. In contrast, approaches that the ability to control behavior by ceding control over thought content, or approaches that reduce cognitive fusion between thought content and behavioral responses, should help to reduce the aversive reactions associated with physiological responses or ruminative thought patterns. To this end, the use of values-based, goal-directed approaches central to the individual’s life goals would be important as a motivating force. Because these individuals will tend to focus on worrisome thought content, focusing on salient, values-based concerns external to the individual are key. If they are to worry, then the focus should be on productive worries, such as staying healthy out of obligations for their role in the family, out of deference to their primary care physicians’ wishes, or as a means of maintaining autonomy in older age.

Several key intervention modalities might also be helpful in this patient population, including modifying the focus of self-monitoring and control over contextual factors. In other words, *the profile of behavior change techniques in this phenotype would* var*y such that attention and perceived control is focused externally for self-monitoring and internally focused for selection of contextual factors, such as location and modality of training*. Behavioral regulation is thereby shifted away from SN aversive stimuli to ECN controllable stimuli. Because these individuals are generally too attentive to physiological cues and/or aversive sensations, finding self-monitoring processes that emphasize external progress markers (over which they have control) are preferred. For these individuals, finding environmental contexts in which the individual has higher perceived control is also critical.

*Example 3 (**Anchored**: high emotion regulation, low cognitive flexibility)*: For individuals with low neuroticism, who are open to experiences, and are conscientiousness but lack a high level of cognitive flexibility, intervention targets are focused on strategy, cuing, and environmental modifications, not on enhancing motivation or affective targets. For these individuals, internal motivational factors and emotional regulation capacity will largely be intact, but their behavioral regulation will often be disorganized or inconsistent. In contrast to affective targets in example 2, most etiologies resulting in low cognitive flexibility are remediated through compensatory techniques, without the expectation that the underlying deficits will improve. Intervening on an individual level with such individuals is often frustrating and fruitless because *these individuals know what they need to do at a content level, but are often unable to execute by deploying these skills when needed to act in context to act effectively, often referred to as an intention-action discrepancy* (Chevance et al., 2018; Pfeffer and Strobach, 2021; Dorina et al., 2023).

In many cases, these deficits result from disruptions between SN-ECN connections with a specific type of phenotypic result: reinforcement learning is blunted, reinforcement cuing is therefore not readily internalized, and the individual therefore relies heavily on the use of external cues to implement action intentions. This pattern is well-known to parents of adolescents, for whom external guardrails and reinforcement strategies are critical until the adolescent is able to slowly internalize behavioral regulation processes, eventually shedding the need for external contingencies. Among older adults, the same profile of deficits results in the need for a gradual reincorporation of external prompts (e.g., reminder systems, white boards for daily tasks, organizer systems), which are often physical modifications to their home environment and leverage visual cues. This is not surprising as the need for prompting (e.g., SN activation) is needed to elicit a strategy-based response, often subserved by posterior ECN brain circuits.

In contrast to adolescents, older adults hold a vast amount of rich life experiences that inform behavioral strategies to their advantage. For this reason, they may often have sophisticated appropriate strategies to deal with behavioral self-regulatory deficits but fail to optimally implement these actions in the appropriate context. This ‘gap’ between capacity and performance often typifies executive dysfunction and is largely remediated by strategies that change the point of cuing for reinforcement learning and action implementation from internal to external. For such individuals, the primary strategy is to narrow the behavioral context in the service of self-regulatory behavioral enhancement and reduce behavioral friction to action implementation. There are diverse examples of how this might be accomplished depending on the behavioral target. Simple examples include hiring a personal trainer instead of self-initiating physical activity, moving in exercise bike into the home and putting it in front of the television or other high-traffic area of the home. Eating healthier, pre-prepared meals or abdicating food preparation responsibilities, where possible, can sometimes mitigate the impact of organizational deficits on poor food choices. Above all else, cultivating routines built around behavioral activation cycles is paramount. As detailed in work on habit formation and maintenance (Wood and Neal, 2016), the more reproducible the daily routine the less mental energy it requires to implement.

In addition to having more inefficient learning for new behavioral skills, Anchored individuals will have a much higher likelihood of gravitating back toward prior coping styles during the maintenance phase of behavioral change. Instantiating changes that they can maintain over time will take longer and they are at a greater likelihood of not following through, even when motivated and accepting of the emotional distress needed to make change. For these individuals, making changes that bolster their social support or alter their physical environment are more critical and should be left in place for longer in order to affect long term change. These are individuals for whom linking them with social structures to facilitate activity is critical, such as getting involved in a local walking club, enrolling in an elderly center nearby, or yoking their activity to other highly ingrained activities (e.g., walking instead of driving to the diner the eat at daily). In addition, the more complex the behavior change, the longer and more gradual their behavioral adaptation will usually take. These are individuals for whom their risk of stopping at target behavior between completing brief rehabilitation and transitioning to maintenance care is very high.

*Example 4 (**Scattered**: low emotion regulation and low cognitive flexibility)* Individuals in this group will be the hardest to intervene with due to a convergence of both deficits. Given the central importance of organization and structure to achieving behavioral targets, *targeting social support system resources external to the individual is likely to be the most effective initial strategy*. As the individual gains more facility over using behavioral response pattern targets, they may benefit more from enhancing motivational factors or otherwise optimizing psychological targets. The integral role of both cognitive and psychological factors has been demonstrated perhaps most compellingly among older adults participating in rehabilitation interventions, for whom executive function moderates treatment engagement and ultimately intervention efficacy (Nes et al., 2011; Ercal et al., 2021). In chronic pain settings, for example, beneficial effects on self-management have not only been demonstrated but appear to be mediated by improved psychological flexibility (Solberg Nes et al., 2009, 2010). These benefits, however, are contingent on adequate engagement with the intervention and, among populations with more variable or limited executive functioning, may require greater involvement from social support members (Caes et al., 2021). Within the BCRM framework, this class of interventions is referred to as facilitating, as it is focused on the provision of external resources instead of strengthening internal reflective resources (boosting) or activating internal resources (nudging) (Michaelsen and Esch, 2022).

Interventions should therefore plan to elicit a greater amount of external support for a longer period of time. Interventions focusing on behavioral changes, such as physical activity, are critical early on as these help to both improve mood and enhance cognitive functions. Mood would also need to be prioritized over and above any cognitive compensatory strategies, as lower mood will remain a barrier to the learning and deployment of new skills. Skills to assist with emotion regulation have been widely reviewed elsewhere and include exposure-based approaches, stress inoculation, affect labeling, cognitive reappraisal, and cognitive bias modification (Tabibnia, 2020). As the individual gains confidence and self-efficacy, compensatory strategies can be gradually introduced (Kwasnicka et al., 2016). As with Anchored individuals, external supports will need to remain in place for an extended period of time and often indefinitely in order to facilitate maintenance behaviors.

## 5 Discussion

Our proposed framework draws upon the overarching construct of self-regulation to propose that behavioral interventions may work optimally when they align with individual- and social systems-level factors that mediate and moderate personal goal pursuit across aging. Differences at either level may impact the efficacy of behavioral interventions and should be considered when designing and implementing complex behavioral treatments. The proposed treatment paradigm is intended as a starting point for further elaboration and not as a fully developed, prescriptive conceptual framework. On the contrary, we hope that the proposed conceptual paradigm will be useful in identifying heterogeneity across treatment frameworks and potential areas were targeted intervention.

Although our proposed paradigm is in need of additional empirical validation, we note there are multiple previously published examples that accord with the proposed framework. An extensive literature has explored the relationships between personality and treatment engagement, generally demonstrating that individuals who are more open, conscientious, and less neurotic are better able to engage effectively in treatment (Bucher et al., 2019). Executive functioning is increasingly conceptualized as both a predictor and consequence of behavior change (Hofmann et al., 2012, 2014; Lopez et al., 2017; Dohle et al., 2018). While empirical work verifying these complex associations is still emerging, recent data in both the rehabilitation (Ercal et al., 2021) and weight loss literature (Butryn et al., 2019) support the notion that baseline differences may play an important role in predicting behavioral trial outcomes. Similarly, prior randomized trials of exercise training have demonstrated that treatment-related improvements in executive function predict subsequent exercise maintenance (Best et al., 2014), leading some to propose a possible ‘virtuous cycle’ by which physical activity and cognition may have reinforcing effects (Audiffren and Andre, 2019). Finally, a wealth of data suggests that social support plays an important role in treatment outcomes, although fewer studies have attempted to integrate the understanding of social support and individual-level predictors within behavioral trials. For example, within our own prior clinical trials we have demonstrated that behavioral modification conducted with a clinical psychologist can improve clinical outcomes among individuals with heart failure to modify lifestyle and coping behaviors (e.g., physical activity, salt intake, etc.) (Sherwood et al., 2017). In secondary analyses of treatment improvements, however, we found that higher social support potentiated the effects of coping skills, such that the effects of treatment were much more pronounced among individuals with higher levels of social support who presumably had greater opportunities to both learn and deploy behavioral skills (Blumenthal et al., 2019). Although few studies have attempted to integrate social support measures to understand their potential effect as a treatment buffer, numerous trials (including our own) have suggested that treatment efficacy is often moderated by baseline markers of disease burden (Smith et al., 2010; Blumenthal et al., 2014; Sherwood et al., 2016; Smith et al., 2022b).

Put simply, the proposed paradigm attempts to operationalize what clinicians already know: treatments work best when they are tailored for the patient and their clinical needs. The present paradigm therefore attempts to codify this clinical intuition in order to help guide clinicians as to which patient characteristics to prioritize when delivering behavioral interventions and how to integrate among seemingly desperate patient- and systems-level factors. Patients who have greater cognitive flexibility to navigate behavioral changes, as well as those whose personalities lend themselves to a broader diversity of changes, will be more likely to navigate change successfully. Among those with inflexibility, either in their cognitive capacity or temperamentally, social support may be important to help them navigate the complexities of behavior change in the most efficient manner. Finally, these considerations are particularly important among individuals with greater medical burden, either as indicated by comorbidities or rapidly evolving treatment requirements (e.g., introduction of multiple medications). Individuals lacking adequate social support or the individual capacity to handle significant change are those who may preferentially benefit from structural resources to help optimize outcomes (e.g., structured rehabilitation paradigms, nursing-led medication adherence paradigms).

Future studies could attempt to test the proposed model in several ways. First, studies should continue to incorporate measures of personality and executive functioning when possible, in order to better refine individual differences and chronic disease self-management. Emerging data strongly suggest that both factors play an important role in determining self-management and behavioral outcomes (Rhodes and Quinlan, 2015; Sloan et al., 2017; Audiffren and Andre, 2019; Gowey et al., 2021), although few studies have attempted to integrate both factors (Joyner and Loprinzi, 2018). Prior work has suggested the use of several approaches to better characterize the efficacy of different coping responses. These include richer collection of process-level data that includes (1) specificity of target strategy, (2) how much regulatory effort is exerted, (3) how many different strategies are used, (4) whether the individual perseverated on ineffective strategies, and (5) how often individuals return to previously abandoned strategies (Aldao, 2013). Assessments of personality, in particular, are widely available and have been shown to have acceptable psychometric properties, making them easier to integrate within clinical research settings. Moreover, the integration of both factors is consistent with emerging resilience frameworks that hypothesize a central role for affect regulation in promoting resilience behaviors (Troy et al., 2023).

Second, future studies should more systematically assess social support in order to examine its influence on maintenance of self-management behaviors, particularly among complex patient populations where the burden of medical compliance or symptomatic burden is higher (Smith et al., 2018; Blumenthal et al., 2019). It is also likely that social support plays an important role during transitions from different behavioral intervention treatment phases, particularly from the ‘active’ (and often supervised) intervention periods to maintenance phases (which are typically unsupervised). Assessments of social support should include measures of both structural and perceived social support (Hailey et al., 2023). Perceived social support measures commonly used include the Perceived Social Support Scale and UCLA Loneliness scale (Hailey et al., 2023). Additional more structural measures of support could include social isolation (Hawthorne, 2006), identifiable social support (Shah et al., 2022), functional social support (Cohen et al., 1985), and social network density (Lubben, 1988). At a minimum, investigators and clinicians should ask about whether the individual at the focus of treatment lives alone and, if so, how available (both geographically and in terms of time) other caregivers are (Sweegers et al., 2019; Fearnbach et al., 2021; Tsuji, 2022; Tanaka et al., 2023). A related and increasingly available metric to assess structural support are emerging area deprivation indices, which provide information on environmental impoverishment that appear to predict health behavior outcomes over and above family income levels (Steppuhn et al., 2019; Melo et al., 2021; Congdon, 2022; Marshall et al., 2023; Witkam et al., 2023).

Third, future studies should continue to examine psychological flexibility as a key mechanism linking psychological and cognitive factors to behavioral outcomes, given its emerging importance within both chronic disease and neurological patient populations (Faulkner et al., 2020; Aytur et al., 2021; Faulkner et al., 2021). If empirically validated, the proposed framework would provide important data to guide personalized treatment approaches to enhance self-management behaviors in older adults. Finally, future studies should consider collecting data on individual response patterns during times of both low and high demand, for example immediately following exercise training. Data on how individuals are experiencing and coping with higher demands of physical activity and/or weight loss could provide important data to tailor treatment approaches that optimize the use of coping skills (Ekkekakis, 2017; Dunton et al., 2023; Liu et al., 2023).

## Author contributions

PS: Conceptualization, Writing – original draft, Writing – review & editing. HW: Writing – original draft, Writing – review & editing. RMM: Writing – original draft, Writing – review & editing. CO’H: Writing – original draft, Writing – review & editing. TS: Writing – original draft, Writing – review & editing.
